# Evidence for a Dopamine Intrinsic Direct Role in the Regulation of the Ovary Reproductive Function: *In Vitro* Study on Rabbit Corpora Lutea

**DOI:** 10.1371/journal.pone.0104797

**Published:** 2014-08-22

**Authors:** Francesco Parillo, Margherita Maranesi, Fiorenzo Mignini, Lisa Marinelli, Antonio Di Stefano, Cristiano Boiti, Massimo Zerani

**Affiliations:** 1 Scuola di Bioscienze e Medicina veterinaria, Università di Camerino, Matelica, Italy; 2 Dipartimento di Scienze biopatologiche veterinarie ed Igiene delle produzioni animali e alimentari, Sezione di Fisiologia, Università di Perugia, Perugia, Italy; 3 Scuola del Farmaco e dei Prodotti della salute, Università di Camerino, Camerino, Italy; 4 Dipartimento di Farmacia, Università “G. D'Annunzio”, Chieti, Italy; Clermont Université, France

## Abstract

Dopamine (DA) receptor (DR) type 1 (D1R) has been found to be expressed in luteal cells of various species, but the intrinsic role of the DA/DRs system on corpora lutea (CL) function is still unclear. Experiments were devised to characterize the expression of DR types and the presence of DA, as well as the *in vitro* effects of DA on hormone productions by CL in pseudopregnant rabbits. Immunoreactivity and gene expression for D1R decreased while that for D3R increased in luteal and blood vessel cells from early to late pseudopregnant stages. DA immunopositivity was evidenced only in luteal cells. The DA and D1R agonist increased *in vitro* release of progesterone and prostaglandin E2 (PGE2) by early CL, whereas the DA and D3R agonist decreased progesterone and increased PGF2α *in vitro* release by mid- and late CL. These results provide evidence that the DA/DR system exerts a dual modulatory function in the lifespan of CL: the DA/D1R is luteotropic while the DA/D3R is luteolytic. The present data shed new light on the physiological mechanisms regulating luteal activity that might improve our ability to optimize reproductive efficiency in mammal species, including humans.

## Introduction

Dopamine (DA) is a catecholamine neurotransmitter that is extensively distributed in the brain and also in different peripheral organs of numerous species [1]. The physiological effects of DA are produced through its interaction with specific DA receptors (DR), which are G protein-coupled receptor sites; in mammals, there are five subtypes of receptors, grouped in the superfamilies of D1R-like (called D1R and D5R in human and D1A and D1B in other mammals) and D2R-like (D2R, D3R, and D4R) receptors [1], [2]. Recently, Yamamoto *et al.* [3] have proposed a new classification for the D1 receptor class suggesting that ancestral jawed vertebrates possessed four classes of D1 receptor genes (D1A, D1B, D1C, and D1E): the D1C receptor gene went secondarily lost in the mammalian lineage, whereas the D1E receptor gene was lost independently in several lineages of modern vertebrates. Generally, D1R-like receptors stimulate the production of the second messenger cAMP, whereas D2R-like receptors dampen the production of cAMP, resulting in a decrease of protein kinase A activity [2].

In mammals, dopamine receptors are expressed in several organs and tissues, such as the nervous system, gastrointestinal tract, kidney, cardiovascular system, and reproductive system [2]. Within the ovary, D1R protein was identified also in luteal cells of human [4], [5], horse [6], rat [7], and cow [8]. These findings suggest that DA may exert a novel physiological regulatory pathway directly involving corpora lutea (CL) function. Corpora lutea are transient endocrine gland that result from rupture of ovulatory follicles and secretes progesterone, a hormone which is necessary for the development and maintenance of successful pregnancy [9]. It is widely accepted that prostaglandins (PG) play a key role in regulating CL function and life span. PGF2α is the main luteolysing factor, while PGE2 is the pivotal luteoprotective factor with luteotrophic and/or antiluteolytic actions [10]–[14].

The renaissance of the rabbit as an animal model for biomedical studies in the toxicology of pregnancy and for the developmental origins of health and diseases [15] together with the possibility of inducing timely pseudopregnancy through administration of exogenous gonadotropin-releasing hormone (GNRH) [16], suggest that rabbit CL offer a good model for investigating the possible role of the DA/DRs system in regulating luteal function. Therefore, the main objectives of the present study were to examine in rabbit CL, during early, mid, and late luteal stages of pseudopregnancy, the gene expression of DRs and DA and their immunolocalization as well as the *in vitro* effects of DA on the production of progesterone, PGE2, and PGF2α.

## Materials and Methods

### Reagents and hormones

Goat polyclonal anti-D1R (sc-31479), -D3R (sc-7525) -D5R (sc-1441), mouse monoclonal anti-D4R (sc-13169), and -D2R (sc-5303) primary antibodies were obtained from Santa Cruz Biotechnology (Santa Cruz, CA, USA); mouse monoclonal anti-DA primary antibody was purchased from Thermo Scientific (Fremont, CA, USA). Biotin horse anti-goat and goat anti-mouse IgG secondary antibodies were obtained from Vector Laboratories (Burlingame, CA, USA). The avidin-biotin complex (ABC, Vector Elite Kit), the chromogen 3,3′-diaminobenzidine tetrachloride (DAB) and horse- and mouse-IgG were purchased from Vector Laboratories. Reagent for isolation of total RNA (TRIzol) was purchased from Invitrogen (S. Giuliano Milanese, Milano, Italy). iSCRIPT cDNA and iQ SYBR Green SuperMix were purchased from Bio-Rad Laboratories (Hercules, CA, USA). The QIAquick PCR Purification Kit for sequencing PCR product was from Qiagen (Milano, Italy). Real-time PCR primers for D1R, D3R, and 18S were supplied by Invitrogen. Gene Ruler 100 bp DNA Ladder was from Fermentas (Thermo Scientific). Tritiated hormones and were purchased from Amersham Biosciences (Amersham Biosciences Ltd, Little Chalfont, Bucks, UK), while progesterone, PGF2α, and PGE2 antisera, and non-radioactive hormones came from Sigma (St Louis, MO, USA). Incubation wells were obtained from Becton Dickinson Co (Clifton, NJ, USA), while medium 199 and Earles Balanced Salt Solution were from GIBCO (Grand Island, NY, USA). HEPES and BSA were purchased from Sigma, whereas all other pure grade chemicals and reagents were obtained locally. Dopamine hydrochloride, Dihydrexidine hydrochloride, SCH 23390 hydrochloride, 7-Hydroxy-PIPAT maleate, and GR 103691 were purchased from Tocris Bioscience (Bristol, UK), AH 6809, AH 23848, and AL 8810 from Cayman Chemical Company (Ann Arbor, MI, USA). The following hormonal preparations were administered via i.m. injection: PMSG (Folligon, Intervet, Milan, Italy) and GnRH analogue (Receptal, Hoechst-Roussel Vet, Milan, Italy). Protein concentration was determined by Bio-Rad Protein Assay Kit (Bio-Rad Lab).

### Animals and hormonal regimen

The protocols involving the use of the animals for these experiments were approved by the Bioethic Committee of the University of Perugia. Sexually mature New Zealand white female rabbits (n = 24, 3.5–4.0 kg body weight and 5 months of age), raised in premises owned by the University of Perugia, were used for all experiments. The rabbits (*Oryctolagus cuniculus*) were housed individually in wire mesh cages under controlled light (14L∶10D; lights off at 2100 h) and temperature (18–24°C) conditions. Each animal had free access to food and water. Pseudopregnancy was induced with 20 IU eCG followed 3 days later by 0.8 µg buserelin [15]. The day of buserelin injection was designated day 0. At day 4 (early stage), 9 (mid), and 13 (late) of pseudopregnancy, animals (n = 5 for each luteal stage) were sacrificed by cervical dislocation in accordance to the guidelines and principles for the care and use of research animals.

### Tissue collection and processing

Upon sacrifice, reproductive tracts were promptly removed and thoroughly washed with saline. Within a few minutes, all CL were excised from ovaries and, after careful dissection of non-luteal tissue by fine forceps under stereoscopic magnification, some were immediately processed for *in vitro* experiments while others were rinsed with RNAse free phosphate buffered saline and frozen at −80°C for later evaluation of gene expression. For the immunohistochemical studies, the ovaries of three additional animals for each luteal stage were fixed by immersion in 4% (w/v) formaldehyde in PBS (pH 7.4) for 24 h at room temperature, and subsequently processed for embedding in paraffin following routine tissue preparation procedures [17]. Six brains and kidneys were obtained from additional rabbits to evaluate the specificity of antisera as positive (immunohistochemistry) and negative (Western blotting) reference.

### Immunohistochemistry

Immunohistochemical investigation was performed according to procedures previously described [18]. Slides were deparaffinized and rehydrated through graded concentrations of alcohol to distilled water. Then, they were dipped in 3% H_2_O_2_ diluted in methanol for 1 h to quench the endogenous peroxidase activity and rinsed in TBS. Background labeling was prevented by incubating the sections with normal goat serum (for anti-DA, -D4R, -D2R antisera) and normal horse serum (for anti-D1R, -D3R, -D5R antisera) diluted 1∶10, for 1 h at room temperature. The slides were then incubated overnight at 4°C in a moist chamber with goat-polyclonal anti-D1R, D3R and-D5R (1∶100) and mouse monoclonal anti-DA, -D4R and -D2R (1∶100) primary antibodies diluted in TBS containing 0.2% Triton X-100 and 0.1% bovine serum albumin (BSA). The next day, the slides were rinsed in TBS, treated again with normal goat serum or normal horse serum and incubated with biotin horse anti-goat (for anti-D1R, -D3R, -D5R antisera) and goat anti-mouse (for anti-DA, -D4R, -D2R antisera) secondary antibodies diluted 1∶200, for 30 min at room temperature. After TBS washes, the slides were exposed to avidin-biotin complex (ABC kit) for 30 minutes and rinsed again with TBS. The peroxidase activity sites were visualized using the DAB kit as chromogen; the sections were rinsed with distilled water and counterstained with Mayer's haematoxylin. Then, they were washed in running tap water, dehydrated by passing through graded ethanol (vol/vol: 70, 95 and 100%), cleared in xylene and, finally, mounted with Eukitt medium for light microscopy. Tissue sections in which the primary antibody was omitted or substituted by rabbit or mouse IgG were used as negative controls of non-specific staining. In order to show the specificity of D1R and D3R antibodies, a positive control was included using rabbit brain striatum tissue that was processed for immunoistochemistry as above described. The intensity of immunostaining was assessed and compared microdensitometrically as previously described [19] using an image analysis system (IAAS 2000 image analyzer, Delta Sistemi, Rome, Italy).

### RNA extraction and real-time PCR

Total RNA was extracted from CL of three rabbits for each luteal stage as previously described [12]. Five µg of total RNA was reverse transcribed in 20 µl of iSCRIPT cDNA using random hexamer according to the protocol provided by the manufacturer. Genomic DNA contamination was checked by developing the PCR without reverse transcriptase. Serial experiments were carried out to optimize the quantitative reaction, efficiency, and CT values. The optimal 25 µL PCR reaction volume contained 12.5 µL of iQ SYBR Green SuperMix, 1 µL forward and reverse primers (stock concentration 10 µM), and water to 25 µL. The primers used are listed in Table 1. All reagents were mixed as a master mix and distributed into a 96-well PCR plate before adding 2 µL of cDNA (diluted 10 fold with water). For every PCR run, reaction controls without template, as negative controls, and without reverse transcriptase in RT were included to ascertain that RNA was free of genomic DNA contamination. The amplification fidelity of samples was also verified by agarose gel electrophoresis. PCR was performed on an iCycler iQ (Bio-Rad) with an initial incubation at 95 C for 1.5 min, followed by 40 cycles at 95°C for 15 s, 53°C for 30 s, during which fluorescence data were collected. The threshold cycle (Ct value) was automatically computed for each trace. PCR products were purified and sequenced by Qiaquick PCR Purification Kit according to the manufacturer's protocols. The 18S Ct housekeeping gene was determined to normalize sample variations in the amount of starting cDNA. Standard curves were generated by plotting the threshold value (Ct) against the log cDNA standard dilution (1/10 dilution) in nuclease-free water. The slope of these graphs was used to determine the reaction efficiency. Sample mRNA quantification was evaluated by iCycler system software, while that of mRNA gene expression was calculated by the 2^−ΔΔCt^ method [20]. The melting curve analysis, performed immediately after the PCR end cycle, was used to determine the specificity of each primer set. A melt-curve protocol was performed by repeating 80 heatings for 10 s, from 55°C with 0.5°C increments, during which fluorescence data were collected.

**Table 1:** Primers used to assess mRNA expression of dopamine receptor subtype 1 and 3.

Gene	Product size (bp)	Primers (5′-3′)
*D1R*	155	F-ATCTCTTGGTGGCTGTCTTGG
		R-TACCTGTCCACGCTGATCACA
*D3R*	196	F-CTGCAGACCACCACCAACTA
		R-TGATGGCACAGAGGTTCAAG
*18S*	148	F-CGATCAGATACCGTCGTAGT
		R-TTCCTTTAAGTTTCAGCTTTGC

### Western blotting

Total luteal proteins were extracted from a pool of nine rabbit CL. The CL were homogenized in 1 ml/100 mg wet tissue ice-cold RIPA buffer (PBS containing 1% Igepal CA-630, 0.5% sodium deoxycholate, 0.1% SDS v/v) as described previously [12]. The striatum (positive control) and anterior pituitary (negative control) proteins were obtained as described for luteal ones. After incubation at 4°C for 20 min, the homogenates were centrifuged at 12000×g for 60 min at 4°C. The supernatants were collected and their protein concentrations measured using the protein assay kit with BSA as standard. Equal amounts of proteins (20 µg) were separated by discontinuous 10% SDS-PAGE (w/v) with 4% staking gel (w/v) for 40 min at 200 V and 500 mA, after which proteins were transferred onto a nitrocellulose membrane for 1 h at 100 V and 350 mA. After transfer, non-specific binding of antiserum was prevented by incubation with 0.05% Tween-20 (v/v), 5% nonfat dry milk (w/v), and 5% BSA (w/v) in Tris-buffered saline (TBS). Blocked membranes were then probed with antibody against D1R and D3R (1∶2000) overnight at 4°C. After washing with TBS, the membranes were incubated with HRP-labeled rabbit anti-goat IgG secondary antibody (1∶20000) at room temperature for 1 h. All antibody incubations were performed in TBS containing 5% BSA (w/v) and 0.05% Tween-20 (v/v). After washing with TBS, the immune complexes were visualized using an enhanced chemiluminescence detection kit according to the manufacturer's protocol and exposed to X-Ray film. Blot images were acquired using Quantity One software (Bio-Rad Laboratories).

### High performance liquid chromatography

A previously reported high performance liquid chromatography (HPLC) method with electrochemical amperometric detection (EC) was utilized to measure DA concentration in CL [21]. Tissues from each animal were individually homogenized for 2 min with a Dyna-Mix homogenizer (Fisher Scientific) in 500 µL of 0.05 N perchloric acid solution containing (w/v) 0.064% 1-octanesulphonic acid sodium salt, 0.060% heptanesulphonic acid sodium salt, 0.004% sodium EDTA, 0.010% sodium metabisulphite and 25 ng/ml DHBA as an internal standard. The resulting homogenate was then centrifuged at 4500×g for 20 min and the supernatant was filtered using 0.45 µm Millipore filters. The filtrate was set in a low volume insert vial and a portion was injected directly into the liquid chromatography equipment (10 µl).

Analysis were run on the HPLC system consisting of a Waters 600 controller pump, a Rheodyne 7295 injector with a 10-µl loop and an Antec Leyden Decade II detector; the operating potential was 0.75 V. Separation was achieved on a Phenomenex Kinetex RP-C18 column (4.6×150 mm, 5 µm). The mobile phase consisted of 53 mM citric acid, 43 mM sodium acetate, 0.1 mM sodium EDTA, 1.0 mM 1-octanesulfonic acid sodium salt, and 20% methanol. The mobile phase was filtered and degassed by vacuum. A flow rate of 1.2 ml/min was used in all experiments. DA stock solutions were prepared at a concentration of 1 mg/ml in 0.05 N perchloric acid containing 0.064% 1-octanesulphonic acid sodium salt, 0.060% heptanesulphonic acid sodium salt, 0.004% sodium EDTA, and 0.010% sodium metabisulphite. These standard solutions were freshly prepared every week and stored at 4°C for use right away. DA was identified on the basis of retention time. Measurements were performed in triplicate for each original sample.

### *In vitro* experiments

**Figure 1:**
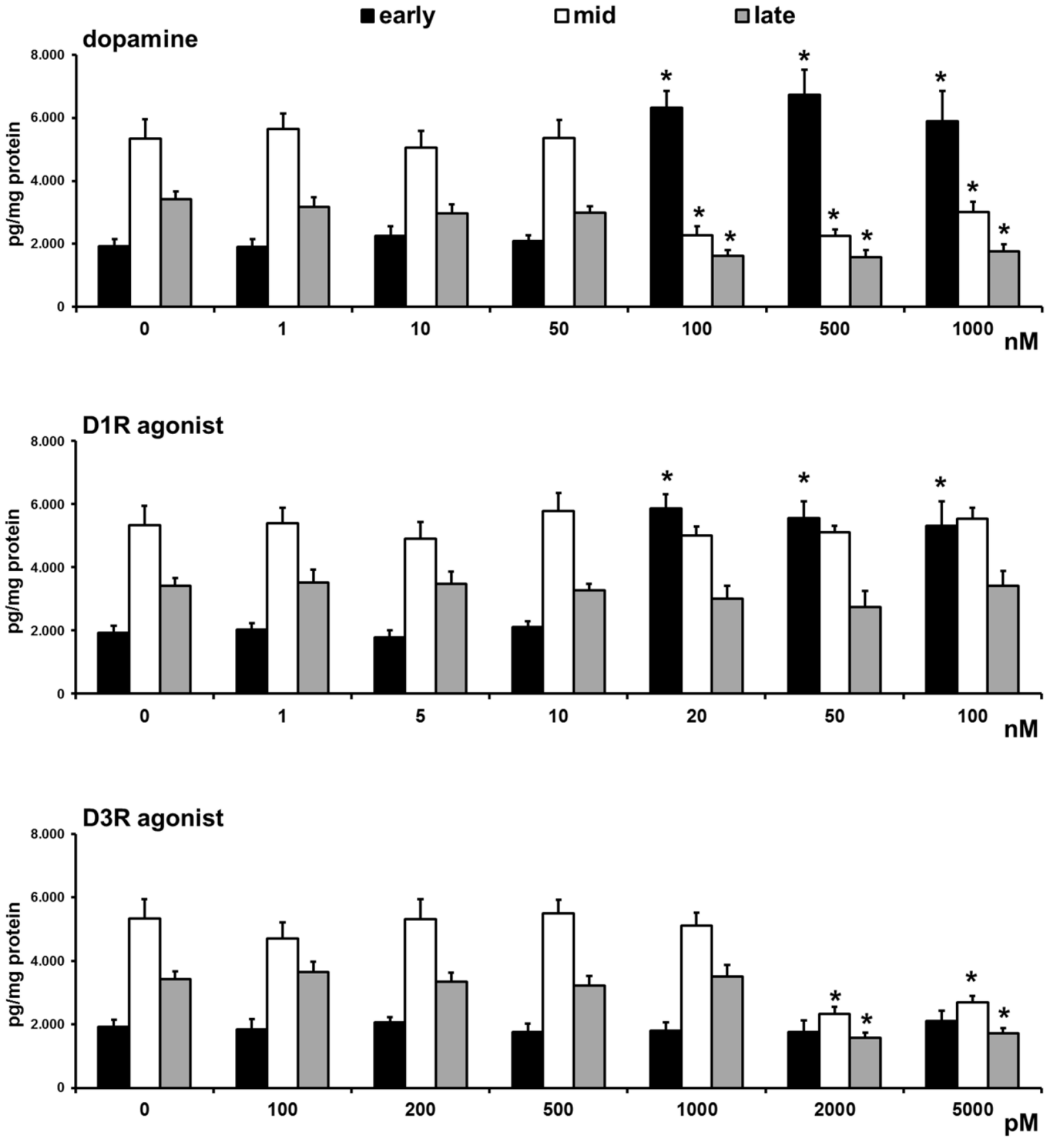
*In vitro* effects of increasing concentrations of dopamine, D1R agonist (dihydrexidine hydrochloride), and D3R agonist (7-hydroxy-PIPAT maleate) on progesterone release by pseudopregnant rabbit CL collected at early, mid, and late luteal stages. Values are the means ± SD of five replicates. Asterisks indicate a significantly different value (P<0.01) versus control (0).

A method previously reported was used for the *in vitro* study [12]. Early, mid, or late CL were randomly distributed (one CL/well) into incubation wells in 1 ml of culture medium 199 with Earles Balanced Salt Solution containing 2.2 mg/ml sodium bicarbonate, 2.3 mg/ml HEPES, and 3% BSA (w/v), referred to here as M199. Before treatment, the CL were quartered inside each well using fine forceps. Each set of incubation wells was divided into 14 experimental groups, each formed of 10 wells as follows: (I) medium alone as control; (II) DA (100 nM), (III) D1R agonist (dihydrexidine hydrochloride, 20 nM); (IV) D1R agonist plus D1R antagonist (SCH 23390 hydrochloride, 1 nM); (V) D1R agonist plus D3R antagonist (GR 103691, 1 nM); (VI) D1R agonist plus PGE2 receptor type 2 (EP2) antagonist (AH 6809, 1 µM); (VII) D1R agonist plus EP4 antagonist (AH 23848, 1 µM); (VIII) D1R agonist plus PGF2α receptor (FP) antagonist (AL 8810, 1 µM); (IX) D3R agonist (7-hydroxy-PIPAT maleate, 2 nM); (X) D3R agonist plus D3R antagonist; (XI) D3R agonist plus D1R antagonist; (XII) D3R agonist plus EP2 antagonist; (XIII) D3R agonist plus EP4 antagonist; (XIV) D3R agonist plus FP antagonist. Since the PGE2 effects on rabbit CL are mediated by the activation of the adenylate cyclase/protein kinase A pathway [22], only the EP2 and EP4 antagonists were used. The culture plates were incubated at 37°C in air with 5% CO2. The media of each well were collected after 4 h of incubation and stored immediately at −20°C for later determination of progesterone, PGF2α, and PGE2. Preliminary evidence led to our choosing the incubation conditions and the minimum effective dose for DA, DA agonists, and DA antagonists used in the present *in vitro* study (Fig. 1).

### Hormone determination

Progesterone, PGF2α, and PGE2 were determined following the RIA protocols previously reported [23]. Intra- and inter-assay coefficients of variation and minimum detectable doses were: progesterone: 6.1%, 8.9%, 11 pg; PGF2α: 7.5%, 12.4%, 18 pg; PGE2: 6.4%, 12.2%, 16 pg.

### Statistical analysis

Data were analyzed by Levene's test and by one-way ANOVA followed by Student-Newman-Keuls t-test. P<0.01 was considered statistically significant.

## Results

### Immunohistochemistry and WB

**Figure 2:**
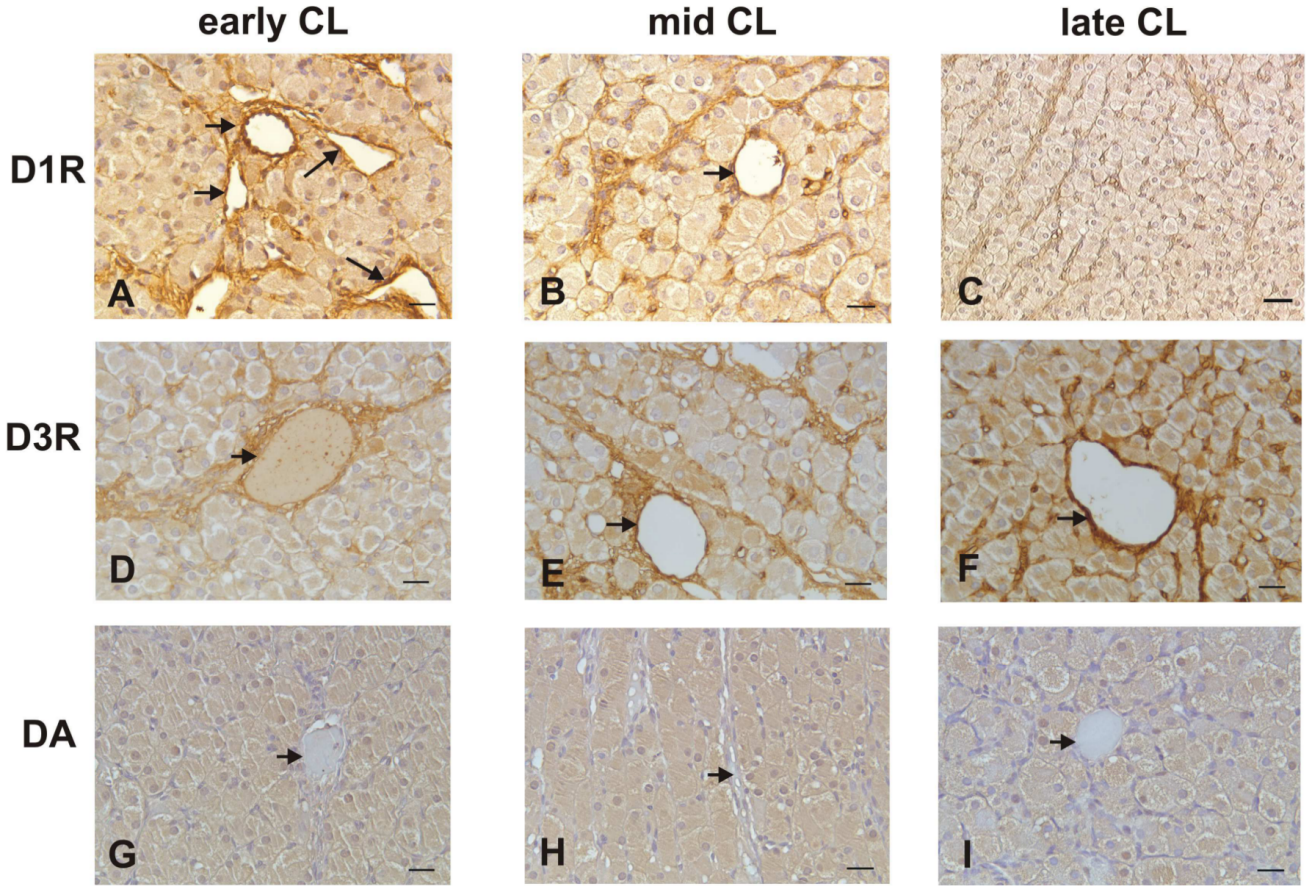
Immunohistochemical demonstration of D1R, D3R, and DA in early, mid, and late CL of pseudopregnant rabbit. A–C: D1R immunoreactivity in luteal and endothelial (arrows) cells. D–F: D3R immunoreactivity in luteal and endothelial (arrows) cells. G–I: DA immunosignals in luteal cells; endothelial cells (arrows) are immunonegative. Scale bars = 20 µm.

**Figure 3:**
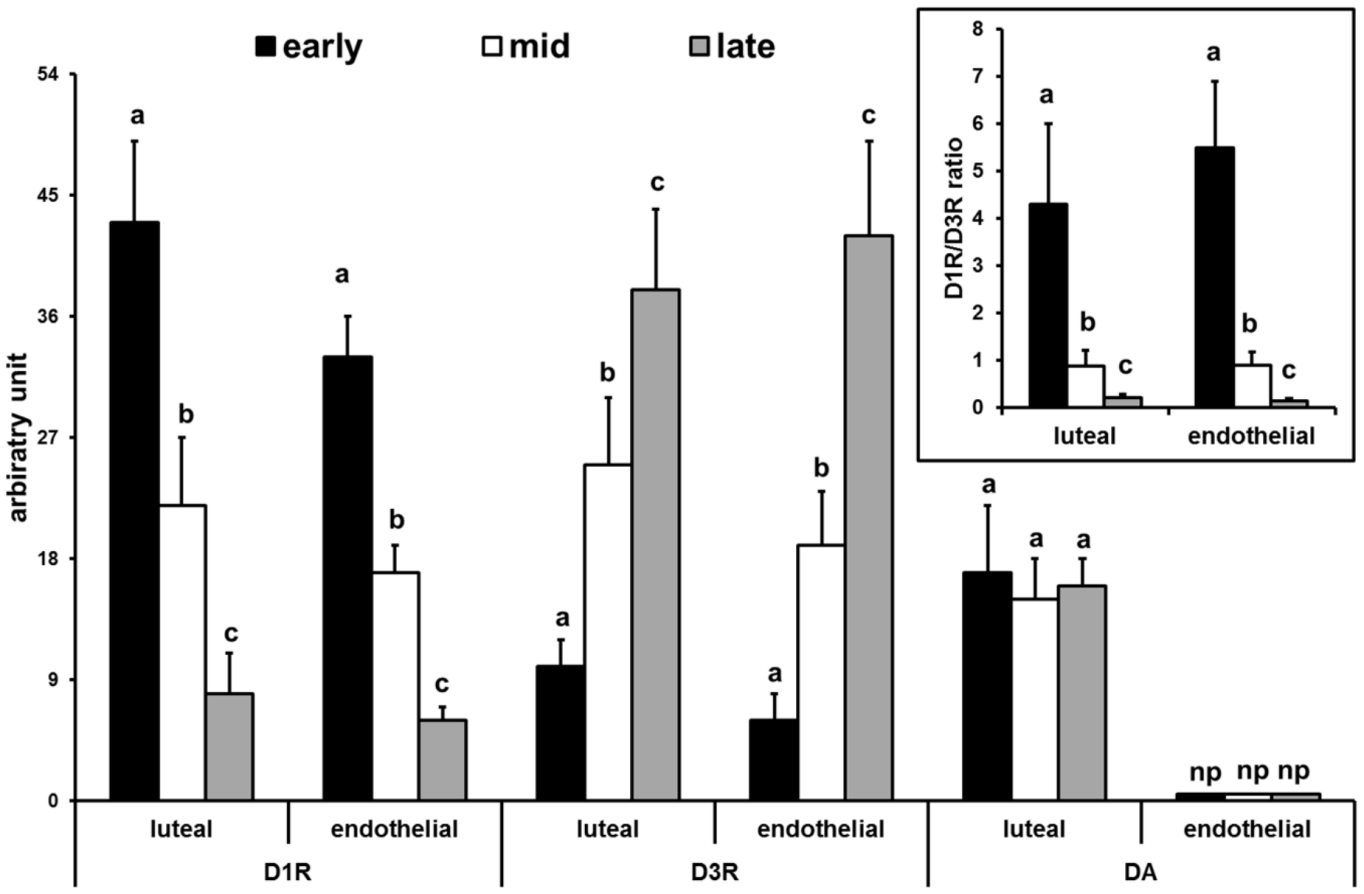
Immunodensitometry of D1R, D3R, and DA in early, mid, and late CL of pseudopregnant rabbit. Insert: D1R/D3R ratio. Data are expressed in arbitrary units; values are the means ± SD of thirty replicates; np: not present; different letters above the bars indicate significant different values (P<0.01).

Positive immunosignals for D1R and D3R were observed in luteal and endothelial cells during all pseudopregnancy stages. In particular, immunoreactivity for D1R decreased (P<0.01) (Figs. 2, A to C, and 3) while that for D3R increased (P<0.01) from early to late stages (Figs. 2, D to E, and 3). The D1R/D3R immunodensitometry ratio of luteal and endothelial cells decreased from early- to late- luteal stage (Fig. 3). D2R, D4R, and D5R were always immunonegative. Positive immune reaction for DA was evidenced only in luteal cells during all stages considered but, differently from D1R and D3R, it did not change among the stages. (Figs. 2, F to G, and 3).

**Figure 4:**
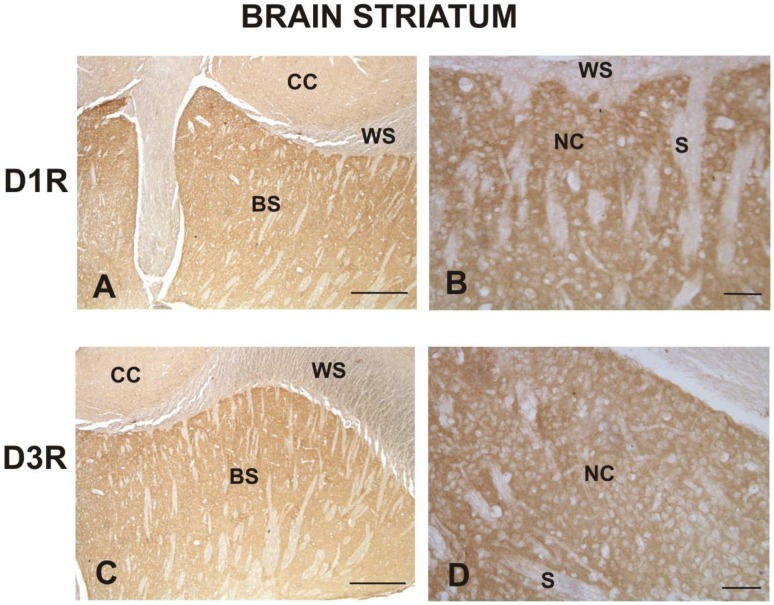
Immunohistochemical presence of D1R and D3R in rabbit brain striatum. D1R (photo A) and D3R (photo B) immunosignals are localized in the cytoplasm of neuronal cells (NC) of the brain striatum. Note the immunonegativity of striosomes (S). Scale bars: photo A, C, E = 200 µm; B, D = 50 µm.

**Figure 5:**
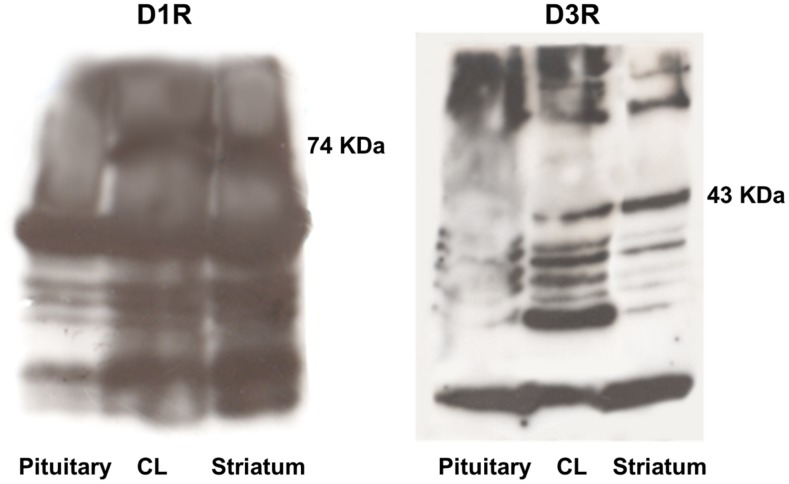
Immunoblot showing D1R and D3R in anterior pituitary (negative control), CL, and brain striatum (positive control) lysates.

**Figure 6:**
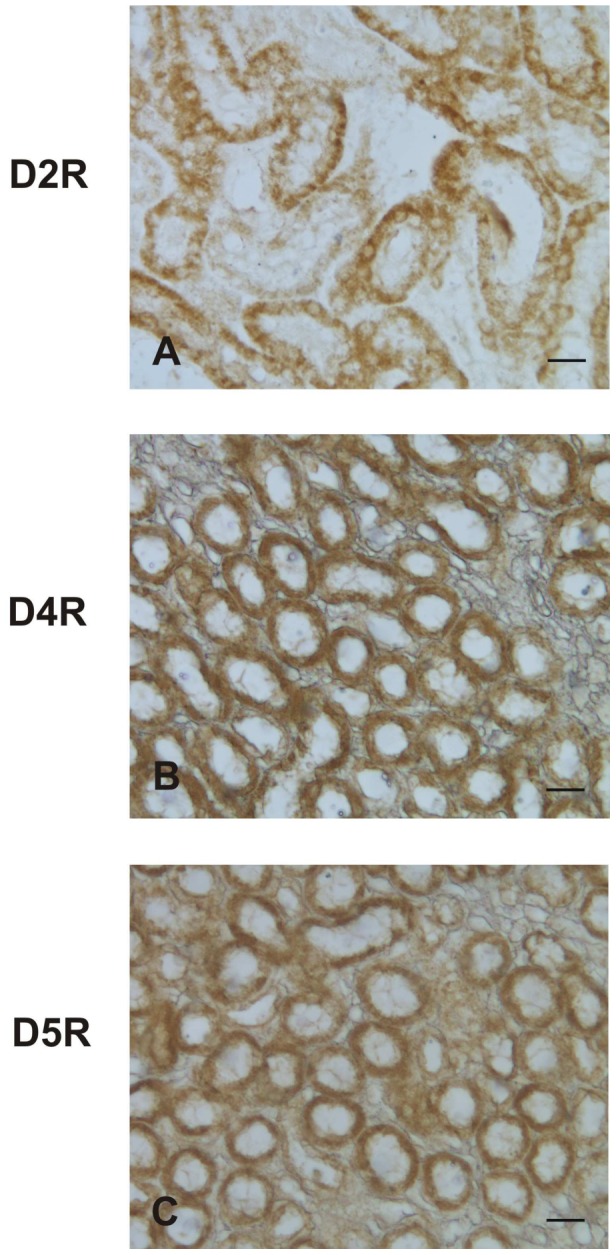
Immunohistochemical presence of D2R, D4R, and D3R in rabbit kidney. Photo A: D2R; photo B: D4R; photo C: D5R. Scale bars: 20 µm.

The brain striatum positive control (Fig. 4) and Western blotting data (Fig. 5) confirmed the specificity of the D1R and D3R antisera used for immunohistochemistry, while the kidney positive control confirmed that of D2R, D4R, and D5R (Fig. 6)

### RT-PCR

**Figure 7:**
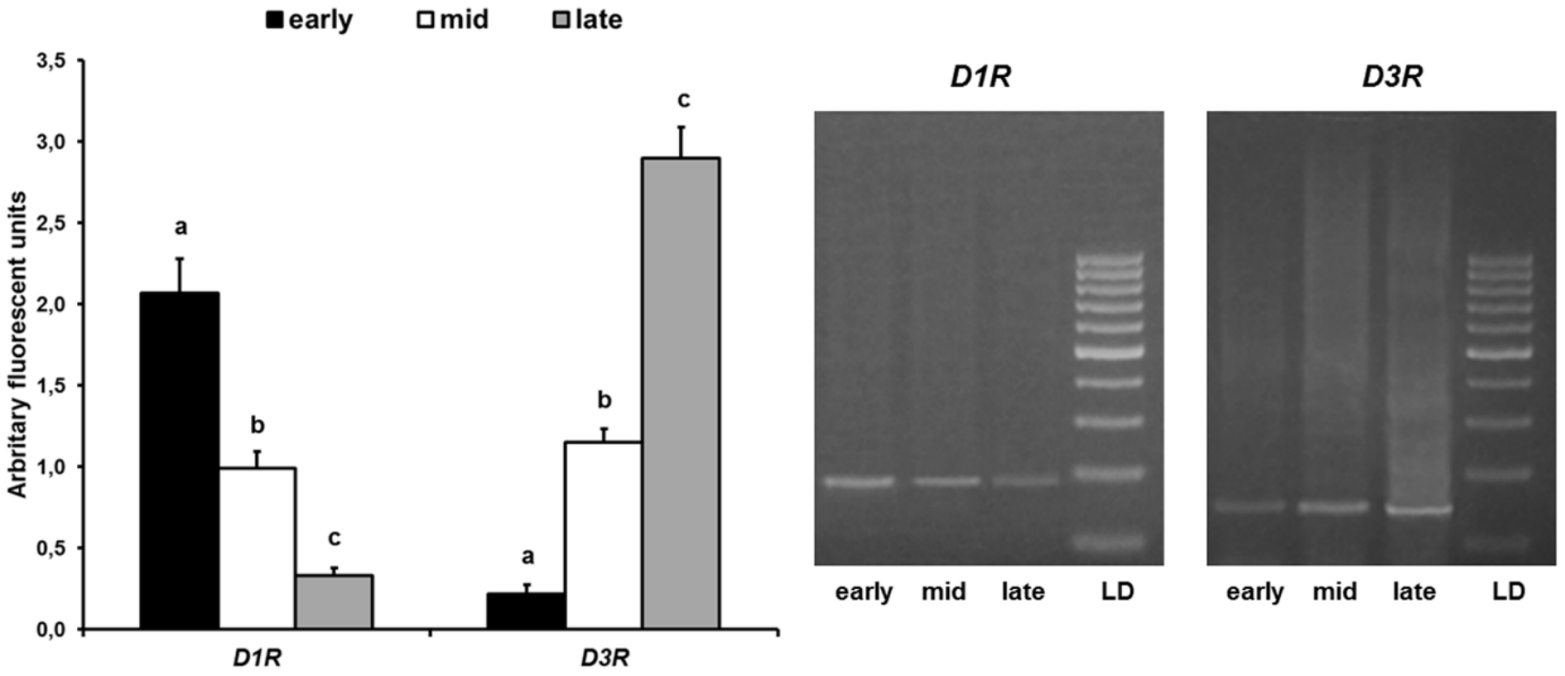
Expression of D1R and D3R mRNA by early, mid, and late CL of pseudopregnant rabbit. Right panel: representative agarose gel electrophoresis stained with ethidium bromide to verify matching between expected and obtained PCR products. Left panel: the values, expressed in arbitrary fluorescence units, are means ± SD for 3 animals/group; different letters above the bars indicate significant different values (P<0.01).

Real-time PCR data of *D1R* and *D3R* showed a comparable trend with that of immunohistochemistry, as mRNA abundance for *D1R* decreased while that for *D3R* increased during pseudopregnancy from early to late luteal stages (Fig. 7).

### HPLC

The concentration of DA did not change during pseudopregnancy (early, 6.53±3.97 ng/mg tissue, n = 5; mid, 3.01±1.82, n = 6; late, 1.17±0,91, n = 5; one-way ANOVA, P>0.01).

### *In vitro* experiment

**Figure 8:**
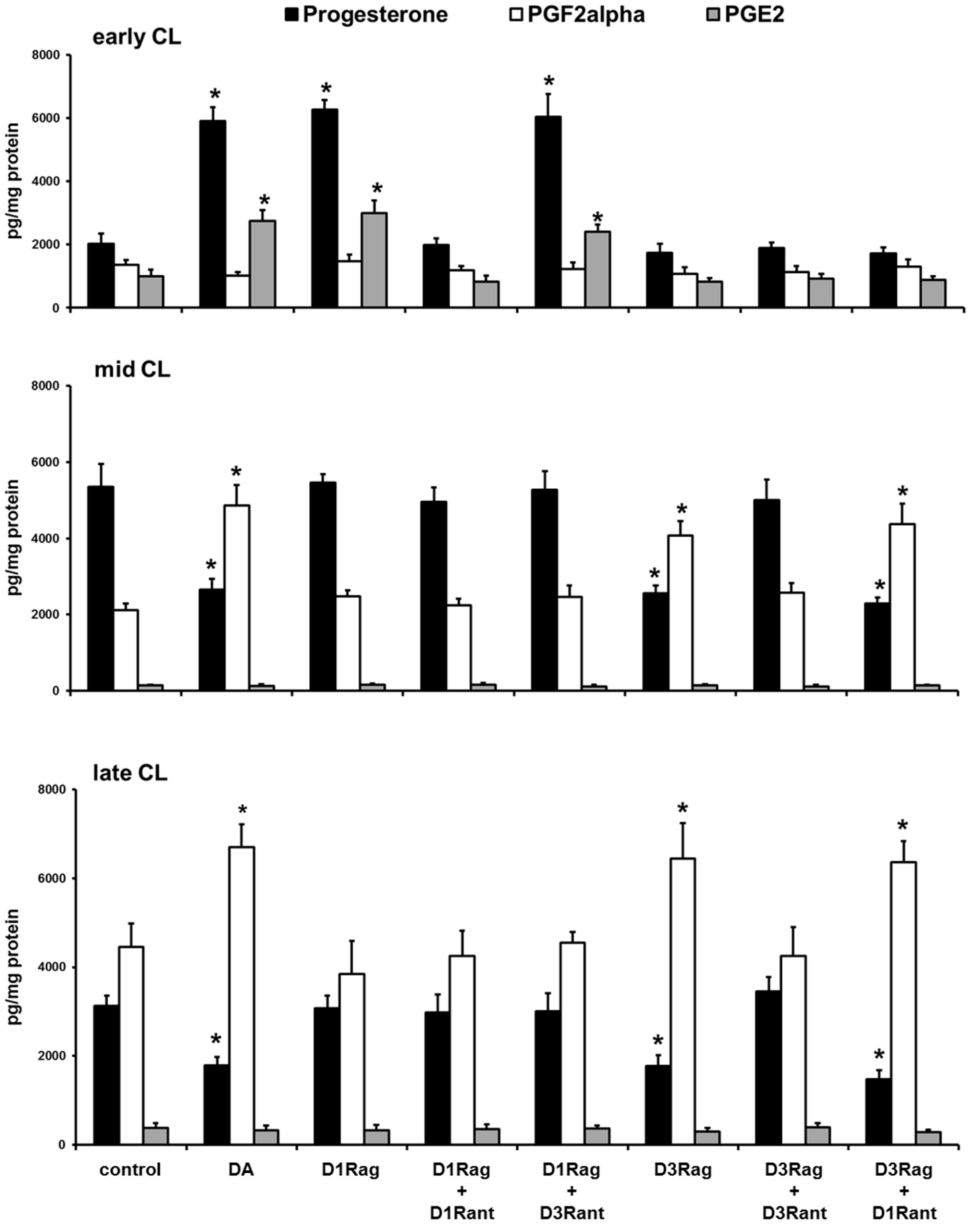
Progesterone, PGF2α, and PGE2 *in vitro* releases by CL of pseudopregnant rabbit. Early (upper panel), mid (middle panel B), and late (bottom panel) CL incubated with DA (100 nM), D1R agonist (D1Rag, dihydrexidine hydrochloride, 20 nM), D1R antagonist (D1Rant, SCH 23390 hydrochloride, 1 nM), D3R agonist (D3Rag, 7-hydroxy-PIPAT maleate, 2 nM), and D3R antagonist (D3Rant, GR 103691, 1 nM). Values are the means ± SD of ten replicates. Asterisks indicate significant different values (P<0.01) versus control.

The *in vitro* results showed that the DA (100 nM) and D1R agonist (dihydrexidine hydrochloride, 20 nM) increased (P<0.01) progesterone and PGE2 production only by early CL, whereas the D3R agonist (7-hydroxy-PIPAT maleate, 2 nM) did not affect this CL type (Fig. 8). On the contrary, the DA and D3R agonist decreased (P<0.01) progesterone and increased (P<0.01) PGF2α production by mid and late CL, whereas the D1R agonist did not affect these two CL types (Fig. 8). Co-incubation with the D1R antagonist (SCH 23390 hydrochloride, 1 nM) counteracted the effects on early CL (Fig. 7), whereas co-incubation with the D3R antagonist (GR 103691, 1 nM) counteracted the effects on mid and late CL (Fig. 8).

**Figure 9:**
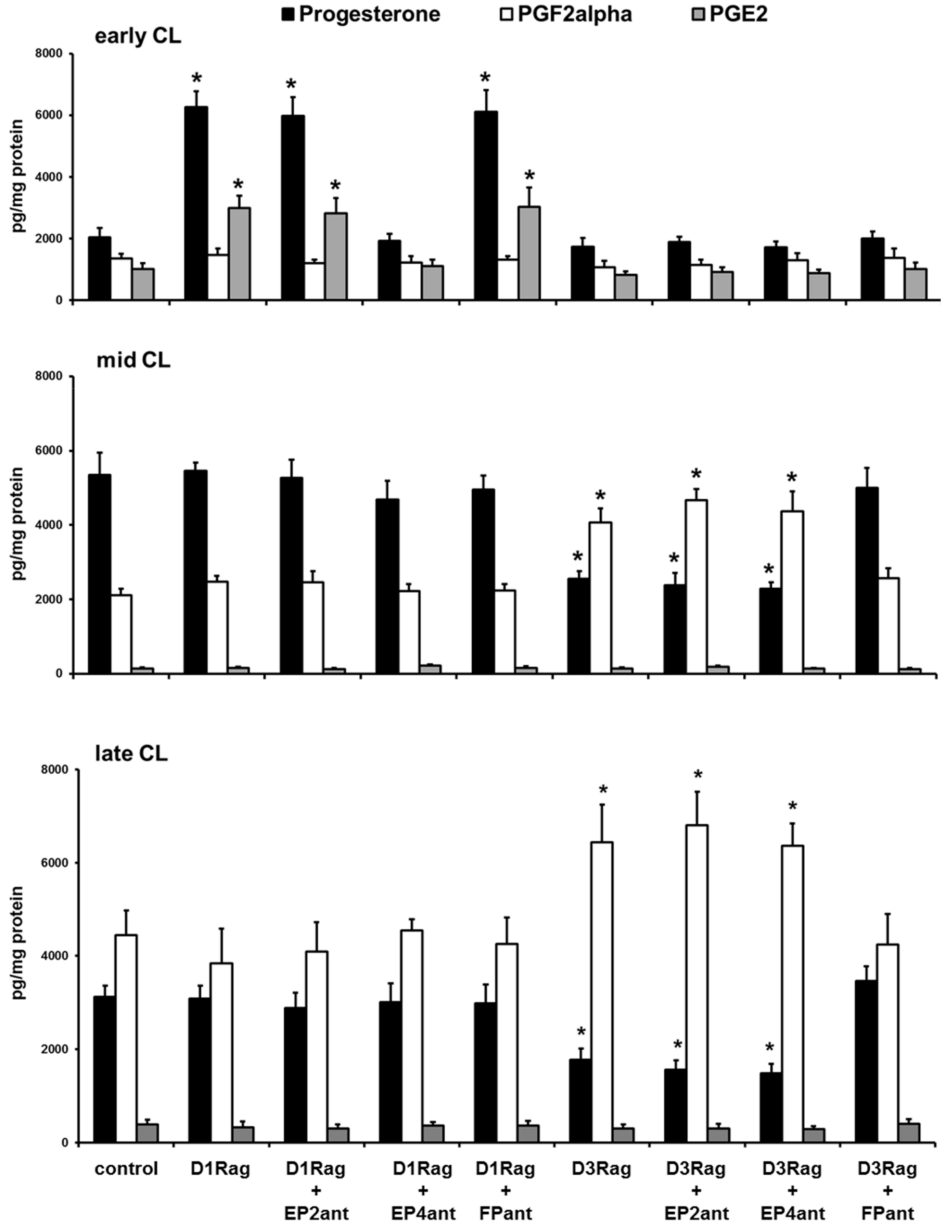
Progesterone, PGF2α, and PGE2 *in vitro* releases by CL of pseudopregnant rabbit. Early (upper panel), mid (middle panel B), and late (bottom panel) CL incubated with, D1R agonist (D1Rag, dihydrexidine hydrochloride, 20 nM), D3R agonist (D3Rag, 7-hydroxy-PIPAT maleate, 2 nM), EP2 antagonist (EP2ant, AH 6809, 1 µM); EP4 antagonist (EP4ant, AH 23848, 1 µM), and FP antagonist (FPant, AL 8810, 1 µM). Values are the means ± SD of ten replicates. Asterisks indicate significant different values (P<0.01) versus control.

Co-incubation with the EP4 antagonist (AH 23848, 1 µM) counteracted the D1R agonist effects on early CL (Fig. 9), whereas co-incubation with the FP antagonist (AL 8810, 1 µM) counteracted the D3R agonist effects on mid and late CL (Fig. 9). Co-incubation with the EP2 antagonist (AH 6809, 1 µM) did not shown any effects (Fig. 9).

## Discussion

To our knowledge, the present study provides the first evidence that the DA/D1R-D3R system has a modulatory, although complex role in controlling the life-span of CL exerting either a luteotropic or a luteolytic action depending on luteal stage. In fact, during the early stage of pseudopregnancy the DA/D1R system promotes *in vitro* PGE2 and progesterone luteal production as demonstrated by the opposite effects induced by specific D1R agonist and antagonist (dihydrexidine hydrochloride and SCH 23390 hydrochloride, respectively), and by the absence of any response for D3R agonist (7-hydroxy-PIPAT maleate) and antagonist (GR 103691).

On the contrary, in both mid and late CL the DA/D3R system showed a luteolytic role by increasing PGF2α production and decreasing that of progesterone. Also in this case, the involvement of D3R was confirmed by the opposite effects in PGF2α and progesterone release following addition of its specific activator to CL of different luteal stages cultured *in vitro*. In addition, the D3R antagonist blocked the effects of DA, whereas the D1R agonist and antagonist had no effects in mid and late CL.

The aforesaid dopamine effects on progesterone are likely induced indirectly, via the modulation of prostaglandin production. In fact, EP4 antagonist (AH 23848) counteracted the D1R agonist effects in early CL, whereas FP antagonist (AL 8810) blocked those of D3R activator in mid and late CL.

Recently, through *in vitro* studies on CL of pseudopregnant rabbits, we demonstrated that PGF2α and PGE2 affected progesterone release differently, depending on the luteal stage [22]. In fact, PGE2 increased progesterone production in the early CL, but was totally ineffective in mid and late CL, while in contrast, PGF2α induced functional luteolysis in day 9 CL, but had no effect on CL collected at the early luteal phase. This physiological mechanism protects the growing CL from functional luteolysis to occur in the early luteal stage until day 6 (a borderline day between early and mid luteal stages) of pseudopregnancy, when CL shift from refractoriness to partial responsiveness to exogenous PGF2α, and acquire luteolytic competence [22], [24], [25]. Other mammalian species such as cows [9], pigs [26], mares [27], and monkeys [28] develop luteolytic capacity at different stages of the estrous cycle. The mechanisms that drive the acquisition of luteolytic competence, however, are still unclear, but recently it has been hypothesized that in rabbits this luteolytic-protecting mechanism also includes the GNRH/GNRH receptor system [29].

Interestingly, the results of the present research suggest that the DA/D1R-D3R system is also probably involved in the shift from luteotropic to luteolytic competence, a change that likely depends on the differential abundance of these two receptors on target luteal cells. In fact, the data on the D1R/D3R protein expression ratio showed that D1R prevails in early CL, whereas D3R is more abundant in both mid and late CL.

After ovulation, newly formed blood vessels cross the basement membrane between theca and granulosa layers and continue rapid growth to sustain CL development and function [9]. The time period of luteal vascular growth varies in cycling and pregnant animals and among species; both angiogenesis and subsequent angioregression are finely regulated by systemic and local factors [30]. Accordingly, our data regarding blood vessel immunoreactivity indicate that the DA/D1R-D3R system may modulate the angiogenesis/angioregression processes in CL, thus supporting our hypothesis about the dual function of DA. In fact, the increase of D3R, a D2R-like receptor, in blood vessels from early to late luteal stages suggests that DA might be involved in luteolysis via luteal angioregression, as found by Sarkar et al. [31] in tumors through its cognate receptor D2R. By converse, the prevalence of D1R over D3R in blood vessels of early CL suggests that the DA/D1R system may act as a luteotropic agent by inducing luteal angiogenesis, although this hypothesis is not yet supported by experimental evidence.

The constitutive expression of DA in the luteal cells during all pseudopregnancy stages supports the possible role for this catecholamine in modulating the CL lifespan, via autocrine and/or paracrine mechanisms for luteal cells and via paracrine for endothelial ones.

The mammalian ovary is connected with the central nervous system through a multisynaptic neural pathway [32]. In addition, intrinsic neurons (neuron-like or neuroendocrine cells) are present in the ovarian interstitial space [33]. These extrinsic and intrinsic neurons, whose actions on ovarian functions are still unclear [33], could be another paracrine source of DA that may bind its cognate receptors D1R and D3R in luteal and endothelial cells; if so, this would strengthen the idea of a physiological role for DA/DRs system in regulating CL function.

In conclusion, the identification of the DA/DRs system as a new regulator of CL lifespan might have important implications for our understanding of ovarian physiology, opening the way to optimize reproductive function and treat reproductive dysfunction in both humans and domestic mammals.
